# Review of Ethnomedicinal Uses, Phytochemistry and Pharmacological Properties of *Euclea natalensis* A.DC.

**DOI:** 10.3390/molecules22122128

**Published:** 2017-12-02

**Authors:** Alfred Maroyi

**Affiliations:** Medicinal Plants and Economic Development (MPED) Research Center, Department of Botany, University of Fort Hare, Private Bag X1314, Alice 5700, South Africa; amaroyi@ufh.ac.za; Tel.: +27-719600326

**Keywords:** ethnopharmacology, *Euclea natalensis*, herbal medicine, traditional uses, tropical Africa

## Abstract

*Euclea natalensis* is traditionally used as herbal medicine for several human diseases and ailments in tropical Africa. This study reviews information on ethnomedicinal uses, botany, phytochemical constituents, pharmacology and toxicity of *E. natalensis*. Results of this study are based on literature search from several sources including electronic databases, books, book chapters, websites, theses and conference proceedings. This study showed that *E. natalensis* is used as traditional medicine in 57.1% of the countries where it is indigenous. *Euclea natalensis* has a high degree of consensus on abdominal pains, antidote for snake bites, diabetes, diarrhoea, malaria, roundworms, stomach problems, toothache, venereal diseases and wounds. Several ethnopharmacological studies have shown that crude extracts and chemical compounds from *E. natalensis* demonstrated many biological activities both in vitro and in vivo, which included antibacterial, antidiabetic, antifungal, antimycobacterial, antiviral, antioxidant, antiplasmodial, larvicidal, antischistosomal, molluscicidal, dentin permeability and hepatoprotective activities. Future studies should focus on the mechanism of biological activities of both crude extracts and chemical compounds from the species, as well as structure–function relationships of bioactive constituents of the species.

## 1. Introduction

*Euclea natalensis* A.DC. (family Ebenaceae) is traditionally used as herbal medicine to treat several human diseases and ailments in tropical Africa. The wide usage of the species as herbal medicine in tropical Africa has resulted in a major resurgence in interest in the ethnopharmacological properties of *E. natalensis*, a plant species characterized by several uses which are recognized culturally, medicinally and commercially. According to Van Wyk [1,2], the roots of *E. natalensis* have commercial potential as remedies for chest ailments, toothache, bronchitis, pleurisy, asthma, headache, and urinary tract infections, as well as mouth rinses or toothbrush sticks that may be developed into pharmaceutical drugs and health promoting products. The twigs and roots of *E. natalensis* are traditionally used as chewing sticks, toothbrushes and mouthwash for oral hygiene, for cleaning teeth and the gums in the belief that these plant parts benefit the health of the mouth and teeth [3]. In southern Africa, the root or twig of *E. natalensis* is peeled from the end of a small root or twig and the end is chewed to a fibrous brush, with the root changing in colour from white to yellow as it is chewed, imparting a pungent and refreshing taste to the mouth [4]. In East Africa, the twigs are used as toothbrushes and roots of *E. natalensis* are also chewed by women to impart a red colour to their mouths [5]. Research by Van Wyk and Gericke [4] showed that, in southern Africa, the root bark of *E. natalensis* is moistened and applied to the lips as a yellow-brown cosmetic by women. Research by Cunningham [6] revealed that roots of *E. natalensis* are sold as herbal medicines in herbal medicine informal markets in Mozambique and South Africa. Similarly, leaves of *E. natalensis* are sold as herbal medicines in herbal medicine informal markets in Tanzania [7].

*Euclea natalensis* is a widely used as herbal medicine in South Africa and the species is an ingredient of a commercial herbal concoction or formula called *imbiza* [8]. The *imbiza* formula or prescription is in clinical use, sold in informal markets, medicinal herbal markets and pharmacies in South Africa. *Imbiza* is a general term for a class of purgative medicines which affect internal cleansing system, often administered as a vaginal douche, a drink or an emetic [9]. *Imbiza* has also gained popularity in South Africa as an immune booster and as a tonic used to treat and manage various minor and chronic illnesses [10]. *Imbiza* is used as herbal concoction for ailments such as colds, chest infections, skin infections, diabetes, tuberculosis, cancer and symptoms of human immunodeficiency virus (HIV) and acquired immunodeficiency syndrome (AIDS) [11]. Traditional healers in South Africa prescribe *imbiza* for women’s fertility problems, as a blood purifier, scrofula and for chest complaints [8,9]. *Imbiza* is also traditionally used to facilitate pregnancy by preparing the uterus to accept a fetus [9]. Apart from *E. natalensis*, *imbiza* also contain roots of *Polygala fruticosa* P. J. Bergius, *Raphionacme* spp., bulbous roots of *Crinum* spp. and *Cyrtanthus obliquus* (L. f.) Aiton and the root barks of *Zanthoxylum capense* (Thunb.) Harv., *Capparis tomentosa* Lam. and *Rauvolfia caffra* Sond. [8]. While the cultural, medicinal and ethnopharmacological values of *E. natalensis* have received considerable attention in the last 50 years [1,2,3,4,8,12,13,14,15,16,17,18,19,20,21], no attempt has been made to review literature on the medicinal potential, phytochemistry and ethnopharmacological properties. Therefore, the aim of the current review is to comprehensively document information on the botany, medicinal uses, phytochemistry and biological activities of *E. natalensis* to understand its ethnopharmacological value as traditional herbal medicine.

## 2. Research Methodology

To identify relevant information on the botany, medicinal uses, phytochemistry and biological activities of *E. natalensis*, a review was compiled based on scientific literature from various sources including Google Scholar, Web of Science, SciFinder, Scopus, Science Direct, PubMed, Scielo, Springerlink, Google Patents, Espacenet, BioMed Central (BMC) and Medline. The keywords used for identification of relevant data included different scientific name and synonyms, common English names, and the terms: biological activities, medicinal uses, ethnobotany, ethnopharmacology, medicinal, pharmacology, phytochemistry and therapeutic value, *Euclea natalensis, Euclea multiflora, Royena macrophylla*, Natal guarri, Natal ebony and large-leaved guarri. Other relevant scientific publications were obtained from the University of Fort Hare library, Alice campus in South Africa.

## 3. Ethnomedicinal Uses of *E. natalensis*

The different uses of *E. natalensis* are summarized in Table 1, including data about herbal preparation and countries where such practices are applied. Information on phytochemicals is summarized in Table 2 and associated pharmacological properties are discussed separately. Ethnomedicinal uses of *E. natalensis* in Table 1 are validated by bibliography shown in Table 2 and pharmacological properties of the species discussed in Section 5 of the manuscript.

*Euclea natalensis* has been recorded in Angola, Botswana, the Democratic Republic of Congo, Ethiopia, Kenya, Malawi, Mozambique, Namibia, Somalia, South Africa, Swaziland, Tanzania, Zambia and Zimbabwe. The species is found in arid and rocky habitats, termite mounds, dune bush, open grassveld, thickets, forests, forest margins, river banks and swamps, with altitude ranging from sea level to about 1600 m above sea level [5,22,23]. It is a shrub or small to medium-sized dioecious tree. The roots, bark, twigs and leaves of *E. natalensis* exhibit several medicinal applications and used to treat or manage various human diseases and ailments throughout the distributional range of the species (Table 1). The roots were the most used plant parts (83.3%), followed by bark and leaves with 6.7% each, leaf sap (3.3%) and twigs (1.7%). A total of 51 ethnomedicinal uses of *E. natalensis* are documented in the literature (Table 1) from eight countries in tropical Africa. The country with the highest number of ethnomedicinal uses is South Africa with 30 ethnomedicinal uses based on 11 literature records, followed by Tanzania with 15 uses and six literature records, Kenya with 11 uses and five literature records, Mozambique with five uses and five literature records and Malawi with five uses and one literature record (Table 1). Literature records show high degree of consensus for at least 12 major diseases and ailments, which include abdominal pains, antidote for snake bites, chewing sticks or toothbrush, diabetes, diarrhoea, malaria, mouthwash, purgative, roundworms, stomach problems, toothache and venereal diseases (Table 1). In some cases, different parts of *E. natalensis* are mixed with plant parts of other species forming an herbal concoction or formula. For example, in South Africa, roots of *E. natalensis* are boiled with roots of *Capparis tomentosa* and thorns of *Gymnosporia heterophylla* and *Phoenix reclinata* and tied to a sharp instrument which is then stabbed into the chest of a patient suffering from pleurisy and pleurodynia [8,14]. In South Africa, *E. natalensis* is an ingredient of herbal concoction called *imbiza* also containing roots of *Polygala fruticosa, Raphionacme* spp., bulbous roots of *Crinum* spp. and *Cyrtanthus obliquus* and root barks of *Zanthoxylum capense, Capparis tomentosa* and *Rauvolfia caffra* used to purify the blood [8]. Research by Chauke et al. [24] revealed that root decoction of *E. natalensis* is taken orally mixed with roots of *Grewia hexamita* and *Pappea capensis* as remedies for stomach complaints and reproductive problems in women such as infertility and painful menstruation. In Tanzania, root decoction of *E. natalensis* is mixed with other plant species such as *Acacia brevispica, Acacia hockii, Acacia robusta, Aloe secundiflora, Asparagus flagellaris, Capparis fascicularis, Carrisa spinarum, Clerodendrum myricoides, Cymbopogon citratus, Dichrostachys cinerea, Eucalyptus* spp., *Gomphocarpus fruticosus, Harrisonia abyssinica, Kedrostis foetidissima, Mangifera indica, Pennisetum purpureum, Psidium guajava, Punica granatum, Musa* spp., *Sansevieria ehlenbergii, Withania somnifera, Ximenia caffra* and *Zanthoxylum chalybeum* as herbal medicine for amoebic dysentery, opportunistic infections and venereal diseases [25].

## 4. Phytochemistry

Various chemical constituents have been isolated from *E. natalensis*, mainly compounds belonging to naphthoquinone and pentacyclic terpenoids classes (Table 2). Lopes and Paul [46] isolated two pentacyclic terpenoids, betulin **1** and lupeol **2** from the root bark of *E. natalensis*, while Tannock [47] isolated naphthoquinones, namely isodiospyrin **3** and mamegakinone **4** from the same species (Table 2). King et al. [48] isolated natalenone **5** from the root bark of *E. natalensis*, while Ferreira et al. [49] isolated natalenone **5**, 8′-hydroxydiospyrin **6**, euclanone **7**, galpinone **8**, methylnaphthazarin **9** and neodiospyrin **10** from the same species (Table 2). Khan et al. [50] isolated lupeol **2**, mamegakinone **4**, diospyrin **11** and 7-methyljuglone **12** from the root bark of *E. natalensis* while Khan [51] isolated 4,8-dihydroxy-6-methyl-1-tetralone (shinanolone **13**) from the same species (Table 2). Weigenand et al. [52] isolated betulin **1**, lupeol **2**, shinanolone **13**, 20(29)-lupene-3β-isoferulate **14** and octahydroeuclein **15** from the root bark of *E. natalensis*. Similarly, Lall et al. [53] isolated betulin **1**, lupeol **2**, shinanolone **13**, 20(29)-lupene-3β-isoferulate **14**, octahydroeuclein **15** and β-sitosterol **16** from the root bark of *E. natalensis* (Table 2). Van der Kooy [54] isolated isodiospyrin **3**, mamegakinone **4**, neodiospyrin **10**, diospyrin **11**, 7-methyljuglone **12**, shinanolone **13** and 5-hydroxy-4-methoxy-2-nathaldehyde **17** from the root bark of *E. natalensis.* Van der Kooy et al. [55] isolated isodiospyrin **3**, mamegakinone **4**, neodiospyrin **10**, diospyrin **11**, 7-Methyljuglone **12** and shinanolone **13** from root bark of *E. natalensis* (Table 2). Bapela et al. [56] assessed the correlation between plant growth and accumulation of diospyrin **11**, 7-methyljuglone **12** and shinanolone **13** in seeds and seedlings of *E. natalensis*, but the compounds accumulated at variable rates and no trend could be established between their synthesis and seedling growth. Bapela et al. [57] assessed seasonal variation of isodiospyrin **3** and neodiospyrin **10**, diospyrin **11**, 7-methyljuglone **12** and shinanolone **13** from wild plants of *E. natalensis* but no defining pattern was established in the synthesis and accumulation of levels of these compounds within the species. Bapela et al. [58] assessed effect of nitrogen, phosphorus and potassium fertilizers on accumulation of isodiospyrin **3**, neodiospyrin **10**, diospyrin **11**, 7-methyljuglone **12** and shinanolone **13**. A significantly positive correlation was established between the concentration of isodiospyrin **3**, neodiospyrin **10**, diospyrin **11**, 7-methyljuglone **12** and shinanolone **13** with fertilization from field-grown seedlings [58]. Joubert et al. [59] used high performance liquid chromatography (HPLC) to quantify the concentration of diospyrin **11** and 7-methyljuglone **12** in roots of *E. natalensis*. The concentration of diospyrin **11** was higher (about 2750 mg/kg) than the concentration of 7-methyljuglone **12** which was about 450 mg/kg. Joubert et al. [59] argued that the observed variation in naphthoquinones concentration could be due to the age of the roots harvested, wound and environmental or other stress factors. Cooper and Owen-Smith [60] showed that *E. natalensis* leaves contain >5% condensed tannins and therefore impalas and kudus avoided grazing the species due to high tannin content while the leaves of the species were accepted by goats.

## 5. Pharmacological Properties

Extracts from *Euclea natalensis* possess a wide spectrum of pharmacological properties, including antibacterial [25,35,40,50,52,61,62,63], antimycobacterial [17,39,52,54,55,64,65,66,67,68,69,70], antifungal [51,57], antiviral [18,71], antidiabetic [72], antioxidant [67,72], antiplasmodial [73], larvicidal [74], antischistosomal [75], molluscicidal [76], dentin permeability [77,78], hepatoprotective [79], cytotoxicity [17,18,35,67,80] and toxicity [19] activities as outlined below. Some of the documented pharmacological properties of crude extracts and compounds isolated from the species may be responsible for the ethnomedicinal uses of the species indicated in Table 1. However, assessment of the ethnomedicinal uses and documented pharmacological effects of the species show that there is not enough systematic data on phytochemistry and pharmacological effects for the majority of the ethnomedicinal applications of the species.

### 5.1. Antimicrobial Activities

*Euclea natalensis* is widely used as herbal medicine for a wide range of infectious diseases caused by microorganisms. Such diseases or ailments include amoebic dysentery, bronchitis, chest complaints, diarrhoea, sexually transmitted infections, sores, syphilis, toothache, tuberculosis, urinary tract infections, venereal diseases and wounds [7,8,13,16,21,24,25,29,30,38,39,41]. Such wide use of *E. natalensis* against bacterial, fungal and viral infections in traditional medicine prompted several researchers to assess antibacterial, antifungal, antimycobacterial and antiviral activities of crude extracts and compounds isolated from the species. The antibacterial, antifungal, antimycobacterial and antiviral investigations reported mixed results as highlighted in the relevant sub-sections below.

#### 5.1.1. Antibacterial Activity

Khan and Nkunya [40] evaluated antibacterial activities of *E. natalensis* root bark extract against *Escherichia coli* and *Staphylococcus aureus*. The extract was active against *Staphylococcus aureus* exhibiting 15–20 mm inhibition zone. Khan and Nkunya [40] evaluated antibacterial activities of the compounds mamegakinone **4**, diospyrin **11** and 7-methyljuglone **12** isolated from *E. natalensis* roots against *Bacillus anthracis, Bacillus cereus, Clostridium perfringens, Corynebacterium diphtheriae, Escherichia coli, Haemophilus influenzae, Klebsiella aerogenes, Neisseria gonorrhoeae, Pseudomonas aureginosa, Salmonella Heidelberg, Shigella dysenteriae, Shigella flexnerii* and *Staphylococcus aureus*. The compounds were active against most of the bacteria except *Escherichia coli* and *Pseudomonas aureginosa*, with inhibition zone demonstrated by the pathogens ranging from 8 mm to 24 mm [40]. A preliminary antibacterial assay showed that the petroleum ether and chloroform extracts of *E. natalensis* root bark gave an inhibitory zone of 15 mm, at an extract concentration of 0.3 mg/mL against *Staphylococcus aureus* [50]. Lall and Meyer [61] evaluated antibacterial activities of water and acetone root extracts of *E. natalensis* against *Bacillus cereus*, *Bacillus pumilus*, *Bacillus subtilis*, *Enterobacter aerogenes*, *Enterobacter cloacae*, *Escherichia coli*, *Klebsiella pneumoniae*, *Micrococcus kristinae*, *Pseudomonas aeruginosa, Serratia marcescens* and *Staphylococcus aureus.* The water and acetone extracts inhibited the growth of *Bacillus cereus*, *Bacillus pumilus*, *Bacillus subtilis*, *Micrococcus kristinae* and *Staphylococcus aureus* at concentrations ranging between 0.1 mg/mL and 6.0 mg/mL. The water extract did not exert any inhibitory action on Gram-negative bacteria, while the acetone extract showed inhibitory activity at a concentration of 5.0 mg/mL against all the Gram-negative bacteria investigated [61]. Weigenand et al. [52] evaluated antibacterial activities of betulin **1**, lupeol **2**, shinanolone **13**, 20(29)-lupene-3β-isoferulate **14** and octahydroeuclein **15** compounds isolated from root bark of *E. natalensis* against *Bacillus cereus*, *Bacillus pumilus*, *Bacillus subtilis*, *Enterobacter cloacae*, *Escherichia coli*, *Klebsiella pneumoniae, Pantoea agglomerans*, *Pseudomonas aeruginosa*, *Serratia marcescens*, *Staphylococcus aureus* and *Streptococcus faecalis* using the agar plate method with streptomycin sulphate as control. The compound shinanolone **13** showed inhibitory activity against Gram-positive bacterial strains at a concentration of 0.1 mg/mL and 20(29)-lupene-3β-isoferulate **14** showed inhibitory activity against *Bacillus pumilus* at a concentration of 0.1 mg/mL [52]. More et al. [35] evaluated antimicrobial activities of ethanol leaf extracts of *E. natalensis* against oral pathogens, namely *Actinobacillus actinomycetemcomitans*, *Actinomyces naeslundii*, *Actinomyces israelii*, *Porphyromonas gingivalis*, *Prevotella intermedia* and *Streptococcus mutans*, using the disk diffusion method. The extracts were active against the tested bacteria with both minimal inhibitory concentration (MIC) and minimum bactericidal concentration (MBC) values ranging from 1.6 mg/mL to 25 mg/mL [35]. Van Vuuren and Naidoo [62] evaluated antibacterial activities of aqueous and a mixture of methanol and dichloromethane (1:1) leaf extracts of *E. natalensis* against bacterial pathogens associated with urogenital or sexually transmitted infections which included *Gardnerella vaginalis, Neisseria gonorrhoeae, Oligella ureolytica* and *Ureaplasma urealyticum*. All methanol and dichloromethane extracts exhibited noteworthy activities with MIC values ranging from 1.5 mg/mL to 2.0 mg/mL against all tested pathogens. The exhibited antibacterial activities validate the ethnobotanical use of *E. natalensis* as herbal medicine against vaginal discharge in Kenya [30], sexually transmitted infections and syphilis in South Africa [8,13,24,38] and venereal diseases in South Africa and Tanzania [8,13,25]. Sharma and Lall [63] evaluated antimicrobial activities of leaf and root ethanol extracts of *E. natalensis* against pathogenic bacteria, *Propionibacterium acnes* using the broth dilution method with tetracycline as positive control. The extracts showed weak activities with MIC value of 250 µg/mL in comparison to MIC value of 3.1 µg/mL demonstrated by tetracycline, the positive control.

Otieno et al. [25] evaluated antimicrobial activities of root ethanol extracts of *E. natalensis*, and extracts of *E. natalensis* mixed with root extracts of *Carissa spinarum*, *Ximenia caffra* and *Harrisonia abyssinica* against *Escherichia coli, Klebsiella pneumoniae, Pseudomonas aeruginosa, Salmonella typhi, Staphylococcus aureus* and *Streptococcus faecalis* using the disc diffusion assay with ampicillin and dimethylsulphoxide (DMSO) as positive and negative controls, respectively. The multi-plant extracts of *E. natalensis* mixed with *Carissa spinarum*, *Ximenia caffra* and *Harrisonia abyssinica* were more active against all tested microbes with inhibition zones ranging from 18 to 22 mm, MIC values ranging 8.3 ± 0.6 µg/mL to 55 ± 2.6 µg/mL and MBC values ranging from 0.02 mg/mL to 0.335 mg/mL [25]. The single extract of *E. natalensis* showed some activity against *Staphylococcus aureus* only with inhibition zone of 14 mm and MIC value of 55 ± 4.4 µg/mL. These results support use and preference of *E. natelensis* mixed with other plant species as remedy for stomach complaints in South Africa [14,24], amoebic dysentery, opportunistic infections and venereal diseases in Tanzania [25].

#### 5.1.2. Antimycobacterial Activity

Lall and Meyer [39] evaluated antimycobacterial activities of acetone and water root extracts of *E. natalensis* against drug-resistant and drug-sensitive strains of *Mycobacterium tuberculosis* H37Rv using the agar plate method. Acetone and water extracts inhibited the growth of *Mycobacterium tuberculosis* at a concentration of 0.5 mg/mL. Lall and Meyer [39] evaluated the acetone and water extracts using a rapid radiometric method against *Mycobacterium tuberculosis* and obtained a MIC value of 0.1 mg/mL against the strains. In 2001, Lall and Meyer [64] evaluated the antimycobacterial activities of diospyrin **11** isolated from *E. natalensis* against the drug-sensitive and drug-resistant strains of *Mycobacterium tuberculosis* using the radiometric respiratory BACTEC assay. The compound diospyrin **11** was active against *Mycobacterium tuberculosis* with MIC value of 100 µg/mL for all strains. Weigenand et al. [52] evaluated antimycobacterial activities of betulin **1**, lupeol **2**, shinanolone **13**, 20(29)-lupene-3β-isoferulate **14** and octahydroeuclein **15** compounds isolated from root bark of *E. natalensis* against a drug-sensitive strain of *Mycobacterium tuberculosis* H37Rv using a rapid radiometric method with TB drugs, streptomycin and ethambutol as controls. The compound shinanolone **13** showed inhibitory activity against a drug sensitive strain of *Mycobacterium tuberculosis* at a concentration of 0.1 mg/mL [52]. Lall et al. [17] evaluated antimycobacterial activities of crude extracts, the compounds betulin **1**, lupeol **2**, diospyrin **11** and 7-methyljuglone **12** isolated from the roots of *E. natalensis* against *Mycobacterium tuberculosis* both in axenic medium and in a macrophage cell line. Crude extract, diospyrin **11** and 7-methyljuglone **12** isolated from the plant exhibited MIC values of 8.0, 8.0 and 0.5 µg/mL, respectively, against *Mycobacterium tuberculosis* while betulin **1** and lupeol **2** were inactive. The MIC value of 7-methyljuglone **12** against a panel of clinical pan-sensitive and drug-resistant strains of *Mycobacterium tuberculosis* ranged from 0.32 to 1.25 µg/mL [17]. Similarly, Van der Kooy et al. [52] evaluated antimycobacterial activities of isodiospyrin **3**, mamegakinone **4**, neodiospyrin **10**, diospyrin **11**, 7-methyljuglone **12** and shinanolone **13** isolated from root extracts of *E. natalensis* against *Mycobacterium tuberculosis* using the radiometric respiratory BACTEC assay. The MIC values of isodiospyrin **3** (10.0 µg/mL), neodiospyrin **10** (10.0 µg/mL), diospyrin **11** (8.0 µg/mL) and 7-methyljuglone **12** (0.5 µg/mL) compared well to those of the known antimycobacterial drugs ethambutol, isoniazid and rifampicin [55]. McGaw et al. [65] evaluated antimycobacterial activities of root extracts of *E. natalensis* and the compound diospyrin **11** isolated from the species against *Mycobacterium bovis, Mycobacterium fortuitum* and *Mycobacterium smegmatis* using a twofold serial dilution assay with anti-TB drug isoniazid as positive control. The root extracts showed some activities with MIC values ranging from 5.7 µg/mL to 16.3 µg/mL against the tested organisms, and the compound diospyrin **11** also showed some activities with MIC values ranging from 15.6 µg/mL to 62.5 µg/mL against the tested organisms [65]. McGaw et al. [66] evaluated antimycobacterial activities of acetone, chloroform and methanol root extracts of *E. natalensis* and the compounds lupeol **2**, neodiospyrin **10**, diospyrin **11**, 7-methyljuglone **12** and shinanolone **13** isolated from the species against *Mycobacterium bovis, Mycobacterium fortuitum* and *Mycobacterium smegmatis* using a twofold serial dilution assay with anti-TB drug isoniazid as positive control. The plant extracts demonstrated activity with MIC values ranging from 5.7 to 664.1 µg/mL against *Mycobacterium bovis, Mycobacterium fortuitum* and *Mycobacterium smegmatis* [66]. The MBC values were relatively high, ranging from 625 to > 2 500 µg/mL against *Mycobacterium bovis, Mycobacterium fortuitum* and *Mycobacterium smegmatis* [66]. Compound lupeol **2** was inactive against all tested pathogens while neodiospyrin **10**, diospyrin **11**, 7-methyljuglone **12** and shinanolone **13** demonstrated activities with MIC values ranging from 1.6 to 166.7 µg/mL and MBC values ranging from 15.6 to 250.0 µg/mL against *Mycobacterium bovis, Mycobacterium fortuitum* and *Mycobacterium smegmatis* [66]. Lall et al. [67] evaluated in vitro antimycobacterial activities of ethanolic shoot extracts of *E. natalensis* against *Mycobacterium tuberculosis* H37Rv using a 96-well microtitre to determine the minimum inhibitory concentration of the extract. The *E. natalensis* extracts were also evaluated for in vivo antimycobacterial activities in *Mycobacterium tuberculosis* H37Rv infected mice. The MIC value of the extract was found to be 125 µg/mL against *Mycobacterium tuberculosis* compared to MIC value of 0.25 µg/mL exhibited by the positive control isoniazid. The antimycobacterial activities of *E. natalensis* extracts evaluated on *Mycobacterium tuberculosis* (H37Rv) infected Balb/c mice showed substantial decrease in bacterial loads when comparing the infected control group to the treatment groups, showing a decrease in lung homogenate colony forming units from 1.5 × 10^6^ (control) to 7.1 × 10^3^ (drug control) [67]. Therefore, the traditional use of *E*. *natalensis* extract against sores, purulent lesions and skin infections, cough could possibly be attributed to the activities of compounds such as of isodiospyrin **3**, neodiospyrin **10**, diospyrin **11**, 7-methyljuglone **12** and shinanolone **14** against *Mycobacterium tuberculosis.* Evaluation of antimycobacterial activities of naphthoquinone compounds isolated from *E*. *natalensis* [54,68,69,70] seem to suggest that 7-methyljuglone **12** and diospyrin **11** are the most active constituents.

#### 5.1.3. Antifungal Activity

Lall et al. [53] evaluated antifungal activities of the compounds betulin **1**, lupeol **2**, shinanolone **13**, 20(29)-lupene-3β-isoferulate **14**, octahydroeuclein **15** and β-sitosterol **16** isolated from the root bark of *E. natalensis* against *Aspergillus flavus, Aspergillus niger, Cladosporium cladosporioides* and *Phytophthora* spp. *Aspergillus niger* was significantly inhibited by shinanolone **13**, 20(29)-lupene-3β-isoferulate **14** and β-sitosterol **16** at 0.01 mg/mL. Of all the compounds tested, only octahydroeuclein **15** was found to be significantly effective against *Phytophthora* spp. at 0.1 mg/mL. The compounds octahydroeuclein **15** and β-sitosterol **16** significantly inhibited the growth of *Cladosporium cladosporioides* at 0.01 mg/mL [53]. None of the isolated compounds exhibited significant activities against *Aspergillus flavus* at 0.01 mg/mL [53]. Van Vuuren and Naidoo [62] evaluated antifungal activities of aqueous and a mixture of methanol and dichloromethane (1:1) leaf extracts of *E. natalensis* against *Candida albicans*, a pathogen associated with genital candidiasis or thrush. The aqueous and a mixture of methanol and dichloromethane extracts exhibited noteworthy activities with MIC values of 0.5 mg/mL and 3.0 mg/mL against *Candida albicans*, respectively. The exhibited anticandidal activities validates the ethnobotanical use of *E. natalensis* as herbal medicine against vaginal discharge in Kenya [30], sexually transmitted infections in South Africa [24,38] and venereal diseases in South Africa and Tanzania [8,13,25].

#### 5.1.4. Antiviral Activities

Lall et al. [18] evaluated antiviral activities of acetone and water extracts of *E. natalensis* and the compound diospyrin **11** isolated from this species against herpes simplex virus Type 1 (HSV-1). The acetone extract of *E. natalensis* showed moderate antiviral activity against HSV-1, at concentrations of 0.1 to 0.02 mg/mL as shown by the reduction of virus-induced cytopathogenic effects and the protection of cells in a cell viability assay. The compound diospyrin **11** exhibited no inhibitory effects while water extracts exhibited weak activity at a concentration of 0.2 mg/mL which corresponded to a 42% cytopathic effect [18]. Mahapatra et al. [71] evaluated the HIV-1 reverse transcriptase inhibition activities of the compound 7-methyljuglone **12** isolated from the roots of *E. natalensis* and its synthetic derivatives against recombinant HIV-1 enzyme using non-radioactive HIV-RT colorimetric assay. The compound 7-methyljuglone **12** and synthesized compounds exhibited potent inhibitory activities ranging from 70% to 100% at 100 µg/mL [71]. These findings will provide baseline data to future research focusing on correlating the traditional use of *E. natalensis* as herbal medicine for viral infections and the antiviral properties of the species.

### 5.2. Antidiabetic Activities

Nkobole et al. [72] evaluated antidiabetic activities of acetone root extracts of *E. natalensis* by assessing in vitro α-glucosidase and α-amylase enzyme assays. The plant extract demonstrated inhibition of 92.6 ± 0.04% and 74.5 ± 0.04% at 0.2 mg/mL on α-glucosidase and α-amylase, respectively. The compounds lupeol **2** and β-sitosterol **16** isolated from the stem bark of *Terminalia sericea* Burch. ex DC. stem bark were evaluated for antidiabetic activities using α-glucosidase and α-amylase enzyme assays. Compounds lupeol **2** and β-sitosterol **16** inhibitory activities on α-glucosidase with 50% maximal inhibitory concentration (IC_50_) values of 66.5 µM and 66.5 µM, respectively [72]. Against α-amylase, the compounds lupeol and β-sitosterol exhibited moderate activities with IC_50_ values of 140.7 µM and 216.0 µM, respectively. These findings validate the traditional use of *E. natalensis* as herbal medicine for diabetes in Kenya [43] and South Africa [24,42].

### 5.3. Antioxidant Activities

Nkobole et al. [72] evaluated antioxidant activities of acetone root extracts of *E. natalensis* using 2,2-diphenyl-1-picrylhydrazyl radical (DPPH) free radical assay. The DPPH scavenging activity of the plant extract was 94.4 ± 0.01% which was comparable to 95.8 ± 0.01% demonstrated by the control, Vitamin C. Nkobole et al. [72] also evaluated antioxidant activities of the compounds lupeol **2** and β-sitosterol **16** isolated from the stem bark of *Terminalia sericea* using DPPH free radical assay. The compound lupeol **2** demonstrated high radical activity, exhibiting half maximal effective concentration (EC_50_) values of 3.66 µM, which was comparable to the EC_50_ values of 2.52 µM demonstrated by the control, Vitamin C [72]. Lall et al. [67] evaluated antioxidant activities of *E. natalensis* ethanolic shoot extracts using theDPPH free radical assay. The IC_50_ value of the extracts against DPPH free radical was found to be 22.55 ± 2.93 µg/mL against 4.34 ± 0.48 µg/mL exhibited by the control, ascorbic acid [67]. These results obtained by Lall et al. [67] and Nkobole et al. [72] are important as intake of antioxidant rich herbal medicines scavenge free radicals and modulate oxidative stress-related degenerative effects.

### 5.4. Antiplasmodial and Larvicidal Activities

Clarkson et al. [73] evaluated antiplasmodial activities of aqueous, dichloromethane, dichloromethane and methanol (1:1) root and stem extracts of *E. natalensis* against *Plasmodium falciparum* using the parasite lactate dehydrogenase assay. *Euclea natalensis* dichloromethane and methanol (1:1) root and leaf extracts showed promising activities with IC_50_ values of 5.1 µg/mL and 5.3 µg/mL, respectively [73]. The antiplasmodial properties demonstrated by *E. natalensis* imply that the species could be a promising candidate for further investigation as plant-based antimalarial agent. Historically, some of the antimalarial drugs have been derived from herbal medicines or from structures modelled on medicinal plant lead compounds and these include the quinoline-based antimalarials as well as artemisinin and its derivatives. Maharaj et al. [74] evaluated larvicidal activities of roots and stem dichloromethane extracts of *E. natalensis* by exposing the third instar *Anopheles arabiensis* larvae to the extracts with acetone and distilled water as controls. The root and stem extracts exhibited 100% mortality after 48 and 96 h of exposure, respectively [74]. These results provide a scientific basis to the traditional uses of *E. natalensis* as herbal medicine for malaria in East Africa [16], Mozambique [15], Tanzania [7,19] and Zimbabwe [20]. 

### 5.5. Antischistosomal and Molluscicidal Activities

Sparg et al. [75] evaluated antischistosomal properties of crude extracts of *E. natalensis* against the schistosomula of *Schistosoma haematobium* with praziquantel and a culture medium blank as controls. The schistosomula were placed into a culture medium to which the plant extracts were added. *Euclea natalensis* was active, killing 66.7% of the schistosomula worms at a concentration of 3.13 mg/mL. The schistosomula worms that were placed in the culture medium blank survived between 12 and 24 h of exposure while the praziquantel MIC value was 1 µg/mL [75]. Ojewole [76] evaluated molluscicidal activities of *E. natalensis* by exposing adult *Bulinus africanus* and *Biomphalaria pfeifferi* to sublethal and lethal doses of crude and aqueous bark, leaf and twig extracts of the species for a period of 24 h using niclosamide (Bayluscide^®^) (Coating Place Inc., Washington DC, WA, US) as reference molluscicide for comparison. The extracts demonstrated moderate to strong molluscicidal activity with lethal dose 90% (LD_90_) value of 50–100 ppm compared to the positive control, niclosamide (Bayluscide^®^) which killed all the snails at a dose of 1 ppm [76]. These pharmacological evaluations are of importance in the traditional use of *E. natalensis* as remedy for schistosomiasis in South Africa [8,13] and remedy for intestinal worms in East Africa [5,16] and Malawi [28] and South Africa [13,14].

### 5.6. Dental Health

Sales-Peres et al. [77] evaluated the effect of an experimental gel containing *E. natalensis* extract on dentin permeability. The study assessed the in vitro variations in hydraulic conductance of dentin after treatment with *E. natalensis* gel and acidified fluorophosphate gel. The acidified fluorophosphate gel was worse for preventing dentin permeability (90.8%), followed by the control gel (77.1%), and the *E. natalensis* extract was the most effective (66.0%). Therefore, *E. natalensis* presented the most effective action to reduce dentin permeability. Sales-Peres et al. [77] revealed that *E. natalensis* gel not only reduced dentin permeability, but also resisted posttreatment citric acid challenge without changing its permeability. This effect can be attributed to the naphthoquinone compounds present in twigs and roots of *E. natalensis* as the compounds result in the dentin tubule obliteration due to the formation of a protective layer on the teeth [77]. Similarly, Sales-Peres et al. [78] evaluated the effect of *E. natalensis* gel on the reduction of erosive wear with or without abrasion, in enamel and dentin. The authors carried out five-day experimental crossover phases with volunteers wearing palatal devices containing human enamel and dentin blocks. The *E. natalensis* gel was applied in a thin layer in the experimental group and was not applied in the control group. The *E. natalensis* gel caused less wear in enamel in the experimental group than in the control group and a statistically significant value was found for erosion and erosion and abrasion in dentin. Therefore, *E. natalensis* may play a role in the prevention of dentin loss under mild erosive and abrasive conditions [78]. Based on the research findings of Sales-Peres et al. [77,78], *E. natalensis* extracts may be an alternative health product to protect oral health and prevent dental caries, tooth wear and dentinal sensitivity. These findings support the traditional use of the twigs and roots of *E. natalensis* as chewing sticks, toothbrush, mouthwash and remedy for toothache in Kenya [5,30], Mozambique [34,35], South Africa [4,8,13,35,38], Swaziland [32] and Tanzania [5,7,21]. The regular use of *E. natalensis* as chewing sticks, mouthwash or toothbrush might control the formation and activity of dental plaque and therefore reduce the incidence of gingivitis and possibly of dental caries [31]. These findings corroborate the wide application of the twigs and roots of *E. natalensis* as chewing sticks, toothbrush, mouthwash and remedy for toothache in Kenya, Mozambique, South Africa, Swaziland and Tanzania [4,5,7,21,30,32,38].

### 5.7. Hepatoprotective Effects

Lall et al. [67] evaluated in vitro hepatoprotective activities of *E. natalensis* ethanolic shoot extracts on human HepG2 cells. The hepatoprotective activities of *E. natalensis* extracts were tested in vivo using a rat model of isoniazid- and rifampicin-induced hepatotoxicity. *Euclea natalensis* showed a hepatoprotective effect (50% at 12.5 µg/mL) and the ability to increase T-helper 1 cell cytokines Interleukin 12, Interleukin 2 and Interferon α by up to 12-fold and the ability to decrease the T-helper 2 cell cytokine Interleukin 10 fourfold when compared to baseline cytokine production [67]. These findings provide a further scientific basis to the invention relating to the ethanolic extracts from the shoots of *E. natalensis* exhibiting immune stimulatory activity and hepatoprotective activity in vitro and in vivo studies [79]. The ethanolic extract of *E. natalensis* provides an immune stimulatory effect on peripheral blood mononuclear cells, selecting a Th1 immune response over a Th2 immune response. Research by [79] showed that ethanolic shoot extract of *E. natalensis* showed significant in vitro hepatoprotective activities against d-galactosamine and in vivo studies, the extract was nontoxic, acting as hepatoprotectant against the toxic effect of some of the first line drugs.

### 5.8. Cytotoxicity and Toxicity

Lall et al. [17] evaluated cytotoxicity of crude chloroform extract of the roots of *E. natalensis*, diospyrin **11** and 7-methyljuglone **12** by exposing different concentrations of samples to green monkey kidney cells (Vero) and a mouse macrophage cell line, J774A.1. Cytotoxicity results for the Vero cell line showed that the crude extract and diospyrin **11** had 50% maximal inhibitory concentration (IC_50_) values of 64.87 and 17.78 µg/mL, respectively. The concentration of 7-methyljuglone **12** that effected a 90% reduction of growth of *Mycobacterium tuberculosis* Erdman within J774.1 macrophages was 0.57 µg/mL [17]. Similarly, Lall et al. [18] evaluated cell toxicity of root extracts of *E. natalensis* by determining the effect of the crude extracts and diospyrin **11** on the monolayers of primary vervet monkey kidney (VK) cells. The dose of the plant samples that inhibited 50% cell growth (ID_50_) after the incubation period was 0.1 mg/mL and 0.2 mg/mL for acetone and water extracts, respectively. The compound diospyrin **11** exhibited an ID_50_ value of 0.02 mg/mL on VK cells. The water extract from the roots of the plant was the least toxic to cell cultures and inhibited the replication of HSV-1 moderately at a concentration of 0.2 mg/mL [18]. More et al. [31] evaluated cytotoxicity of ethanol leaf extracts of *E. natalensis* using the XTT (sodium 3′-[1-(phenyl amino-carbonyl)-3,4-tetrazolium]-bis-[4-methoxy-6-nitro) benzene sulphonic acid hydrate) assay method. The extracts showed cytotoxicity activity on the Vero cell line with IC50 value of 285.1 ± 4.9 µg/mL [31]. Mahapatra et al. [71] evaluated cytotoxicity activities of the compound 7-methyljuglone **12** isolated from the roots of *E. natalensis* and its synthetic derivatives using the XTT assay method. Cytotoxicity results for the Vero cell line showed that the compound 7-methyljuglone **12** and synthesized compounds had EC_50_ values ranging from 2.5 µg/mL to 36.1 µg/mL [71]. Kishore et al. [80] evaluated cytotoxicity activities of 7-methyljuglone **12** isolated from the root extract of *E. natalensis* and a series of its derivatives on MCF-7, HeLa, SNO and DU145 human cancer cell lines using the XTT method. Most of the 7-methyljuglone derivatives exhibited significant toxicity on HeLa and DU145 cell lines with IC_50_ values ranging from 5.3 µM to 10.1 µM [80]. Lall et al. [67] evaluated in vitro cell cytotoxicity using cell lines (primary peripheral blood mononuclear cells, U937 monocytes and Chang liver cells), acute and sub-acute toxicity were carried out on eight-week-old female Balb/c mice by administering ethanolic shoot extracts of *E. natalensis* orally. During the study conducted on the cytotoxic effect of *E. natalensis* on peripheral blood mononuclear cells, U937 monocytes and Chang liver cells, *E. natalensis* extract showed no cellular toxicity with IC_50_ values ranging from 131.3 ± 1.67 µg/mL to 208.9 ± 10.3 µg/mL. An IC_50_ value below 50 µg/mL has been considered to be moderately toxic and samples with a toxicity value higher than 100 µg/mL have been considered to be non-toxic [67]. During mechanistic studies, the extract showed a 50% inhibition of mycothiol reductase activity at 38.62 µg/mL. During the acute and sub-acute studies, *E. natalensis* exhibited no toxic effect and the 50% lethal dose (LD_50_) was established to be above 2000 mg/kg. The extract was able to reduce the mycobacterial load (1.5-fold reduction) in infected mice. Isoniazid and rifampicin caused significant hepatic damage in rats, and the extract was able to reduce the toxicity by 15% and 40% at 50 mg/kg and 150 mg/kg respectively [67].

Moshi et al. [19] evaluated toxicity of ethanol roots and stem extracts of *E. natalensis* using the brine shrimp lethality test. The extracts were toxic with LC_50_ value of 19.33 µg/mL. These results obtained by Moshi et al. [19] indicate the possibility that *E. natalensis* may be toxic calls for assessment of target-organ toxicity studies.

## 6. Conclusions

*Euclea natalensis* is an important and frequently used herbal medicines in tropical Africa. The species is widely used for human diseases and ailments such as abdominal pains, antidote for snake bites, diabetes, diarrhoea, malaria, roundworms, stomach problems, toothache and venereal diseases. Recent research on *E. natalensis* focused primarily on antimicrobial properties of the crude extracts of the species, naphthoquinone and pentacyclic terpenoid compounds isolated from the species. Literature studies revealed that naphthoquinone and pentacyclic terpenoid compounds isolated from *E. natalensis* such as shinanolone **13** exhibited antibacterial effects [50], shinanolone **13**, 20(29)-lupene-3β-isoferulate **14**, octahydroeuclein **15** and β-sitosterol **16** exhibited antifungal effects [53], isodiospyrin **3**, neodiospyrin **10**, diospyrin **11**, 7-methyljuglone **12** and shinanolone **13** exhibited antimycobacterial effects [17,39,52,55,65,66], diospyrin **11** and 7-methyljuglone **12** exhibited cytotoxic effects [17]. The compound 7-methyljuglone **12** appears to be the most efficient antimycobacterial of all the compounds that have been isolated from *E. natalensis* so far. Any future research on *E. natalensis* should consolidate its ethnomedicinal usage with its phytochemistry and pharmacological effects if ethnopharmacological potential of the species is to be fully realized. Such further research should assess mechanisms of actions, clinical effectiveness and proper dosage for the documented ethnomedicinal uses and associated pharmacological activities. Based on current information, the ethnomedicinal uses and documented pharmacological effects of the species show that there is not enough systematic data on phytochemistry and pharmacological effects for the majority of the ethnomedicinal applications of the species. There is still need for research on phytochemical, bioactive compounds and other medicinal ingredients and minerals that can be used to explain the wide use of *E. natalensis* as herbal medicine in tropical Africa. Future studies should also focus on the mechanism of biological activities and structure–function relationships of bioactive constituents of the species. Likewise, animal studies and clinical studies are to a large degree missing and should be carried out to determine the potential of this plant to be used in human medicine.

## Figures and Tables

**Table 1:** Medicinal uses of *Euclea natalensis* based on ailment categories proposed by Cook [26].

Monotherapeutic Applications	Plant Parts Used	Country	References
**Categories/subcategories**			
**Blood system disorders**			
Blood purification	Root infusion taken orally	South Africa	[27]
**Digestive system disorders**			
Constipation, diarrhoea, enema, purgative, stomach problems	Root infusion taken orally	Kenya, Tanzania, Malawi, Mozambique, South Africa	[7,23,28,29,30,31]
**Genitourinary system disorders**			
Sexual stimulation, urinary tract infections, vaginal discharge	Bark, root infusion taken orally	South Africa, Swaziland, Kenya	[8,30,32,33]
**Infections or infestations**			
Anthelminthic, asthma, bronchitis, chewing sticks, gonorrhoea, hookworm, malaria, mouthwash, rabies, schistosomiasis, scrofulous swellings, sexually transmitted infections (STIs), syphilis, toothache, tuberculosis, venereal diseases, yellow fever	Charred and powdered root, leaf sap applied topically or leaf, root decoction taken orally. Roots or twigs used as chewing sticks, mouthwash or toothbrush.	Tanzania, Ethiopia, Mozambique, Kenya, Zimbabwe, South Africa, Malawi, Swaziland	[4,5,7,8,13,14,15,16,17,18,19,20,23,24,28,30,32,34,35,36,37,38,39,40]
**Injuries**			
Burns, leprosy sores, sores, wounds	Root bark decoction or powder applied topically	East Africa, South Africa	[8,13,14,16,23,41]
**Metabolic system disorders**			
Diabetes	Root decoction taken orally	Kenya; South Africa	[24,42,43]
**Nervous system disorders**			
Epilepsy, hypnotic	Root decoction taken orally or root smoke inhaled	South Africa	[4,44]
**Pain**			
Abdominal pains, earache, headache, splenic swellings, ulcers	Root infusion taken orally or applied topically	Kenya, Malawi, South Africa, Swaziland, Tanzania	[8,13,16,23,28,30,32,40]
**Poisoning**			
Antidote for poisoning, snake bite	Powdered leaf applied topically or root decoction taken orally	Kenya, Malawi	[28,29]
**Pregnancy/birth/puerperium disorders**			
Abortifacient, infertility, menstrual problems, puerperium	Root decoction taken orally	Mozambique, South Africa	[41,44,45]
**Respiratory system disorders**			
Chest complaints	Root decoction applied topically	South Africa	[1,2,8]
**Skin, subcutaneous cellular tissue disorders**			
Skin care	Root infusion applied topically	South Africa	[38]
**Miscellaneous**			
Bad dreams, protective charms	Bark infusions applied topically or leaf decoction taken orally	South Africa, Tanzania	[7,8,41]
**Multi-therapeutic applications**			
Amoebic dysentery	Root infusion taken orally mixed with roots of *Carissa spinarum* L., *Harrisonia abyssinica* Oliv. and *Ximenia caffra* Sond.	Tanzania	[25]
Infertility	Root infusion taken orally mixed with roots of *Grewia hexamita* Burret and *Pappea capensis* Sond. & Harv.	South Africa	[24]
Menstrual problems	Root infusion taken orally mixed with roots of *Grewia hexamita* and *Pappea capensis*	South Africa	[24]
Opportunistic infections	Root infusion taken orally mixed with roots of *Acacia hockii* De Wild., *Acacia robusta* Burch., *Clerodendrum myricoides* R. Br., *Cymbopogon citratus* (DC.) Stapf, *Dichrostachys cinerea* (L.) Wight & Arn., *Eucalyptus* spp., *Kedrostis foetidissima* (Jacq.) Cogn., *Mangifera indica* L., *Pennisetum purpureum* Schumach., *Psidium guajava* L., *Punica granatum* L., *Musa* spp., *Withania somnifera* (L.) Dunal and *Ximenia caffra*	Tanzania	[25]
	Root infusion taken orally mixed with roots of *Asparagus flagellaris* (Kunth) Baker, *Capparis fascicularis* DC., *Harrisonia abyssinica, Sansevieria ehrenbergii* Schweinf. ex Baker and *Ximenia caffra*		
	Root infusion taken orally mixed with roots of *Acacia brevispica* Harms, *Aloe secundiflora* Engl., *Carissa spinarum*, *Dichrostachys cinerea*, *Harrisonia abyssinica, Ximenia caffra*		
	Root infusion taken orally mixed with roots of *Carissa spinarum, Harrisonia abyssinica*, *Withania somnifera*, *Ximenia caffra* and *Zanthoxylum chalybeum* Engl.		
	Root infusion taken orally mixed with roots of *Carissa spinarum*, *Harrisonia abyssinica* and *Ximenia caffra*		
Pleurisy	Root infusion taken orally mixed with roots of *Capparis tomentosa* and thorns of Gymnosporia *heterophylla* and *Phoenix reclinata*	South Africa	[14]
Pleurodynia	Root infusion taken orally mixed with roots of *Capparis tomentosa* and thorns of Gymnosporia *heterophylla* and *Phoenix reclinata*	South Africa	[14]
Stomach problems	Root infusion taken orally mixed with roots of *Grewia hexamita* and *Pappea capensis*	South Africa	[24]
Venereal diseases	Root decoction mixed with roots of *Zanthoxylum chalybeum*	Tanzania	[25]
	Root decoction mixed with roots of *Aloe secundiflora*, *Gomphocarpus fructicosus* (L.) W. T. Aiton and *Harrisonia abyssinica,*		
	Root decoction mixed with roots of *Carissa spinarum, Harrisonia abyssinica, Ximenia caffra*		

**Table 2:** Chemical compounds isolated and characterized from *Euclea natalensis* root bark.

No.	Chemical Compound	Chemical Formula	References
	**Pentacyclic terpenoids**		
**1**	Betulin	CH_30_H_50_O_2_	[46,52,53]
**2**	Lupeol	C_30_H_50_O	[46,50,52,53]
**14**	20(29)-lupene-3β-isoferulate	C_40_H_58_O_4_	[52,53]
**16**	β-sitosterol	C_29_H_50_O	[53]
	**Naphthoquinone**		
**3**	Isodiospyrin	C_22_H_14_O_6_	[47,54,55]
**4**	Mamegakinone	C_22_H_14_O_6_	[47,50,54,55]
**5**	Natalenone	C_22_H_16_O_6_	[48,49]
**6**	8′-hydroxydiospyrin	C_22_H_14_O_7_	[49]
**7**	Euclanone	C_22_H_14_O_7_	[49]
**8**	Galpinone	C_33_H_20_O_9_	[49]
**9**	Methylnaphthazarin	C_11_H_8_O_4_	[49]
**10**	Neodiospyrin	C_22_H_14_O_6_	[49,54,55]
**11**	Diospyrin	C_22_H_14_O_6_	[50,54,55]
**12**	7-methyljuglone	C_11_H_8_O_3_	[50,54,55]
**13**	Shinanolone	C_11_H_12_O_3_	[52,53,54,55]
**15**	Octahydroeuclein	C_22_H_22_O_6_	[52,53]
**17**	5-hydroxy-4-methoxy-2-nathaldehyde	C_12_H_10_O_3_	[54]
